# FPR-1 is an important regulator of neutrophil recruitment and a tissue-specific driver of pulmonary fibrosis

**DOI:** 10.1172/jci.insight.125937

**Published:** 2020-02-27

**Authors:** Jack Leslie, Ben J.M. Millar, Alicia del Carpio Pons, Rachel A. Burgoyne, Joseph D. Frost, Ben S. Barksby, Saimir Luli, Jon Scott, A. John Simpson, Jack Gauldie, Lynne A. Murray, Donna K. Finch, Alan M. Carruthers, John Ferguson, Matthew A. Sleeman, David Rider, Rachel Howarth, Christopher Fox, Fiona Oakley, Andrew J. Fisher, Derek A. Mann, Lee A. Borthwick

**Affiliations:** 1 Newcastle Fibrosis Research Group and; 2 Translational and Clinical Research Institute, Newcastle University, Newcastle upon Tyne, United Kingdom.; 3 Interstitial Lung Disease Clinic, Newcastle upon Tyne Hospitals NHS Foundation Trust, Newcastle upon Tyne, United Kingdom.; 4 Firestone Institute for Respiratory Health, Saint Joseph’s Healthcare and Department of Pathology and Molecular Medicine, McMaster University Hamilton, Hamilton, Ontario, Canada.; 5 MedImmune Ltd., Cambridge, United Kingdom.; 6 Institute of Transplantation, Freeman Hospital, Newcastle upon Tyne, United Kingdom.

**Keywords:** Cell Biology, Immunology, Fibrosis, Neutrophils

## Abstract

Neutrophils are the most abundant inflammatory cells at the earliest stages of wound healing and play important roles in wound repair and fibrosis. Formyl peptide receptor 1 (FPR-1) is abundantly expressed on neutrophils and has been shown to regulate their function, yet the importance of FPR-1 in fibrosis remains ill defined. FPR-1–deficient (*fpr1^–/–^*) mice were protected from bleomycin-induced pulmonary fibrosis but developed renal and hepatic fibrosis normally. Mechanistically, we observed a failure to effectively recruit neutrophils to the lungs of *fpr1^–/–^* mice, whereas neutrophil recruitment was unaffected in the liver and kidney. Using an adoptive transfer model we demonstrated that the defect in neutrophil recruitment to the lung was intrinsic to the *fpr1^–/–^* neutrophils, as C57BL/6 neutrophils were recruited normally to the damaged lung in *fpr1^–/–^* mice. Finally, C57BL/6 mice in which neutrophils had been depleted were protected from pulmonary fibrosis. In conclusion, FPR-1 and FPR-1 ligands are required for effective neutrophil recruitment to the damaged lung. Failure to recruit neutrophils or depletion of neutrophils protects from pulmonary fibrosis.

## Abstract 2

FPR-1 is the primary receptor driving neutrophil recruitment to the injured lung, and absence of FPR-1 or depletion of neutrophils protects from pulmonary fibrosis.

## Introduction

The restoration of functional tissue architecture after injury is critical to ensure the survival of an organism. If an injury persists or is of great enough magnitude, then physiological wound repair becomes pathological, leading to a fibrotic response, a reduction in tissue function, organ failure, and ultimately death (1, 2). Fibrosis is defined by an excessive and dysregulated accumulation of collagen and extracellular matrix (ECM) and can affect any tissue/organ (3).

The effective modulation of the magnitude, duration, and resolution of the inflammatory response is critical to determining the physiological versus pathological response to tissue injury. In response to tissue injury, damage-associated molecular patterns (DAMPs) are released from stressed and dead/dying cells and are critical to the initiation of an inflammatory response. DAMPs encompass a group of heterogeneous molecules that initiate and amplify innate immune signalling pathways by engaging pattern recognition receptors (PRRs) to promote inflammatory pathways (4). N-formyl peptide (fMLF) receptors (FPRs) are a family of PRRs that elicit important regulatory effects in multiple pathological conditions, including inflammation and cancer (5–7). In humans, 3 variants of FPR have been identified (FPR-1 to -3), yet it is FPR-1 that has been most intently studied (5). The principal ligands for FPR-1 are bacterial formylated peptides that are actively secreted by invading pathogens or mitochondrial formylated peptides that are passively released from dead and dying host cells after tissue injury (8, 9). However, an increasing number of alternative, non–formyl peptide ligands for FPR-1 are being uncovered, including cathepsin G (10), annexin-1 (11), and FAM19A4 (12), highlighting the promiscuous nature of FPR-1.

FPR-1 was originally described on human neutrophils, where its activation was shown to trigger a wide variety of functions, including chemotaxis, degranulation, ROS production, and phagocytosis (13, 14). More recent data have shown FPR-1 to be expressed on human monocytes and macrophages (15), bronchial and colonic epithelial cells (16, 17), and fibroblasts/myofibroblasts from several tissues (18, 19). FPR-1 expression was shown to be increased in skin fibroblasts isolated from patients with systemic sclerosis compared with normal subjects and to play an important role in regulating fibroblast to myofibroblast transition in vitro (18). However, the importance of FPR-1 in the initiation and progression of fibrosis is ill investigated and further in vivo work is required to tease out the potential therapeutic benefit of targeting the receptor.

Here, we investigated the role of FPR-1 in the initiation and potentiation of fibrosis in multiple organs (lung, liver, and kidney) by examining differences in fibrogenic responses between C57BL/6 WT and *fpr1^–/–^* mice. Although FPR-1 was found to be dispensable for induced fibrosis in the liver and kidney, absence of FPR-1 protected the lung from bleomycin-induced inflammation and subsequently fibrosis. Moreover, we demonstrate that FPR-1 is crucial for neutrophil recruitment to the lung, whereas, by contrast, neutrophils are recruited to the liver and kidney via FPR-1–independent mechanisms. Experimental depletion of neutrophils afforded a protective antifibrogenic effect in bleomycin-injured animals. Hence, we have discovered that FPR-1^+^ neutrophils play a distinct organ-specific role in fibrosis, being essential for pathological tissue remodeling in the diseased lung.

## Results

### Mice deficient in FPR-1 are protected from bleomycin-induced lung inflammation and fibrosis.

**Figure 1:**
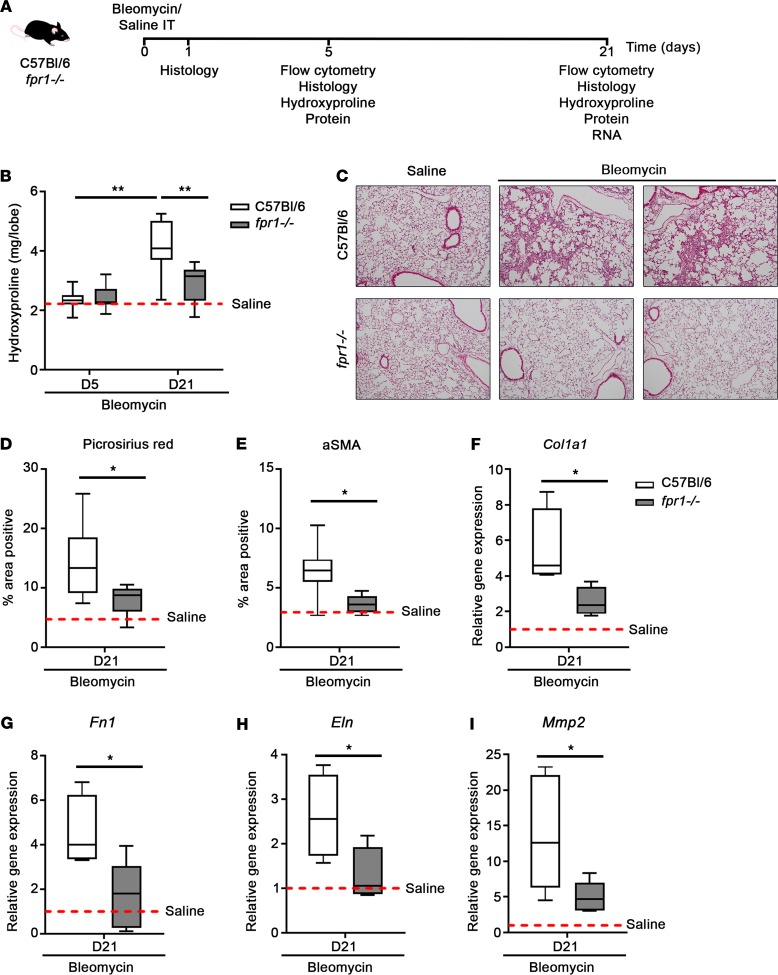
*fpr1^–/–^* mice are protected from bleomycin-induced lung fibrosis. (**A**) C57BL/6 and *fpr1^–/–^* mice were challenged intratracheally with saline (30 μl) or bleomycin sulfate (0.007 U in 30 μl saline) and lung tissue–harvested on days 1, 5, and 21. (**B**) Hydroxyproline (μg/lobe) content of lung tissue on days 5 and 21. (**C**) Representative H&E-stained lung tissue on day 21. (**D**) Percentage area positive of Picrosirius red and (**E**) α-smooth muscle actin (αSMA) staining on day 21. Data represent the mean value of 20 randomly selected, nonoverlapping fields (original magnification, ×20). Relative gene expression of collagen type I (**F**), fibronectin (**G**), elastin (**H**), and MMP2 (**I**) in lung tissue on day 21 was assessed by qPCR. Gene expression was normalized to GAPDH as a loading control. *n* = 7–10 mice per group. No significant difference was seen between saline-treated C57BL/6 and *fpr1^–/–^* mice, and therefore saline-treated mice were pooled and presented as mean (red-hashed line). Data were analyzed using a Mann-Whitney *U* test and presented as box-and-whisker plots. *P < 0.05; **P < 0.01.

WT C57BL/6 and *fpr1^–/–^* mice were challenged intratracheally with saline (control) or bleomycin sulfate (0.007 U) and harvested on days 1, 5, and 21 to assess inflammation and fibrosis (Figure 1A). Fibrosis, as quantified by hydroxyproline content of lung tissue, increased approximately 2-fold (compared with saline) in response to bleomycin challenge in C57BL/6 mice on day 21. Critically, total lung collagen content was significantly lower in bleomycin-challenged *fpr1^–/–^* mice compared with C57BL/6 mice on day 21 (2.89 ± 0.28 vs. 4.11 ± 0.36 μg/lobe; *P* = 0.007) (Figure 1B). Histologically, the extensive tissue remodeling that is classically observed in response to bleomycin challenge was seen in C57BL/6 mice (Figure 1C) and was accompanied by an increase in the percentage area positive for Picrosirius red (collagen type I–III) (Figure 1D) and α-smooth muscle actin (αSMA) (activated myofibroblasts) (Figure 1E). Conversely, *fpr1^–/–^* mice displayed little to no evidence of tissue remodeling and had significantly attenuated collagen type I–III deposition (7.86 ± 0.74 vs. 14.76 ± 2.45%; *P* = 0.014) and myofibroblast activation (3.65 ± 0.22 vs. 6.56 ± 0.86%; *P* = 0.014) compared with C57BL/6 mice. Moreover, *fpr1^–/–^* mice were attenuated for bleomycin-induced expression of genes encoding ECM proteins (collagen type I, fibronectin, elastin) and tissue remodeling enzymes (MMP-2) (all *P* < 0.05) (Figure 1, F–I).

**Figure 2:**
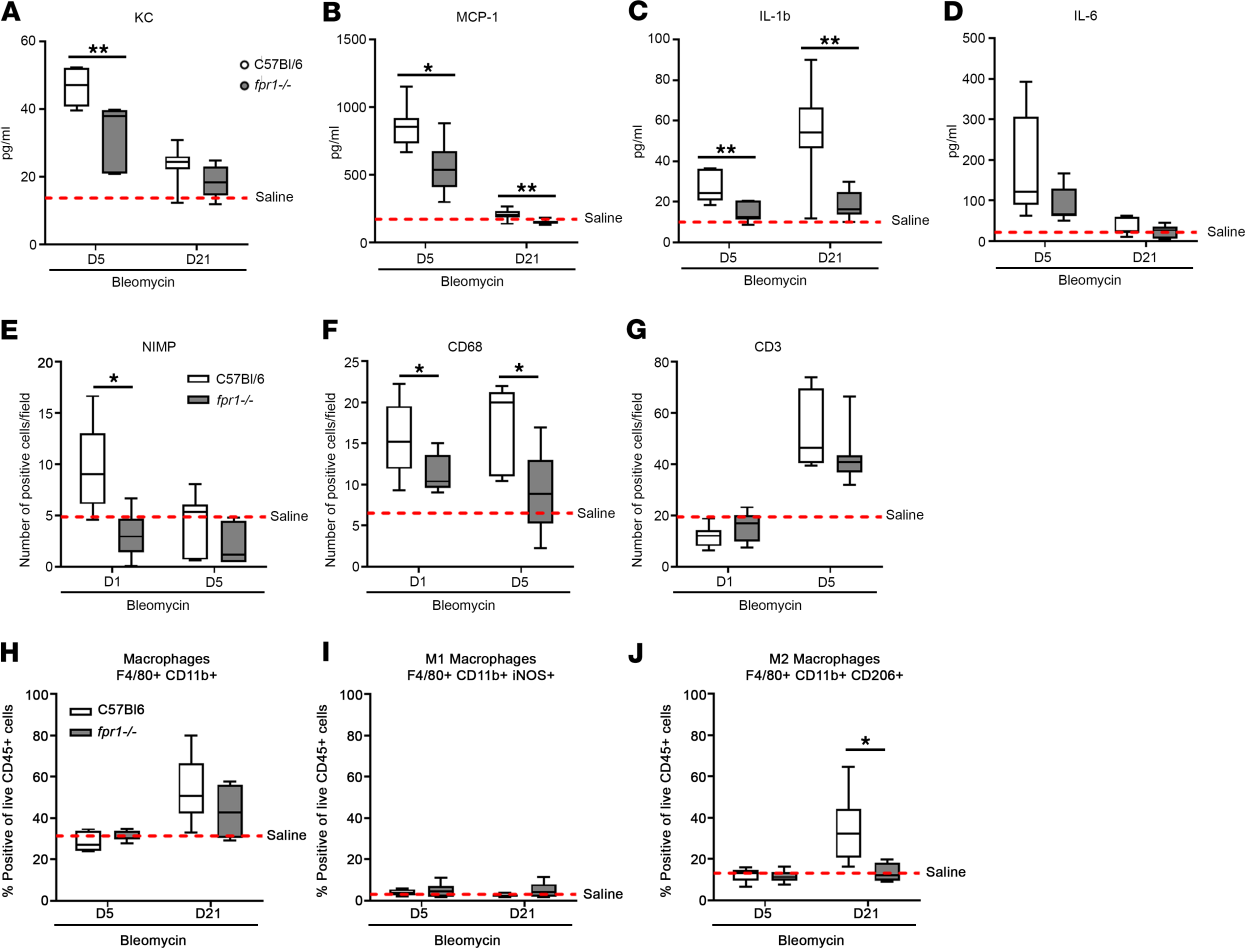
*fpr1^–/–^* mice have reduced inflammation and M2 macrophages in response to challenge with bleomycin. Levels of KC (**A**), MCP1 (**B**), IL-1β (**C**), and IL-6 (**D**) in lung tissue homogenates on days 5 and 21 after bleomycin challenge assessed by multiplex ELISA. Number of (**E**) NIMP^+^, (**F**) CD68^+^, and (**G**) CD3^+^ cells/field in lung tissue on days 1 and 5 after bleomycin challenge. Data represent the mean value of *n* = 20 randomly selected, nonoverlapping (original magnification, ×20) fields per mouse. Frequency of total macrophages (F4/80^+^ CD11b^+^) (**H**), M1 macrophages (F4/80^+^ CD11b^+^ iNOS^+^) (**I**), and M2 macrophages (F4/80^+^ CD11b^+^ CD206^+^) (**J**) in lung tissue were assessed by flow cytometry and presented as a percentage of live CD45^+^ cells. *n* = 7–12 mice per group for A and B. *n* = 4–7 mice per group for **H**–**J**. No significant difference was seen between saline-treated C57BL/6 and *fpr1^–/–^* mice, and therefore saline-treated mice were pooled and presented as mean (red-hashed line). Data were analyzed using a Mann-Whitney *U* test and presented as box-and-whisker plots. *P < 0.05; **P < 0.01.

To assess inflammation in C57BL/6 and *fpr1^–/–^* mice, lung tissue homogenates were prepared on days 5 and 21 after challenge and expression of KC, MCP-1, IL-1β, and IL-6 quantified. In bleomycin-challenged C57BL/6 mice, we observed significant increases in KC, MCP-1, and IL-6 expression on day 5 compared with saline-challenged control mice. On day 21, there was a subsequent reduction back to baseline expression levels for KC, MCP-1, and IL-6. In contrast, although IL-1β was significantly increased on day 5 in bleomycin-challenged C57BL/6 mice, expression peaked on day 21. Expression of KC (31.64 ± 3.51 pg/mL; *P* = 0.001), MCP-1 (565.4 ± 71.8 pg/mL; *P* = 0.018), IL-6 (89.06 ± 16.22 pg/mL; *P* = 0.128), and IL-1β (day 5: 14.71 ± 1.83 pg/mL; *P* = 0.0029; day 21: 18.26 ± 2.03 pg/mL; *P* = 0.0097) was attenuated in *fpr1^–/–^* mice compared with C57BL/6 mice (Figure 2, A–D).

To assess the role of FPR-1 in the recruitment of immune cells into the lung, we quantified the number of neutrophils (NIMP^+^), T cells (CD3^+^), and inflammatory macrophages (CD68^+^) in tissue isolated from challenged C57BL/6 and *fpr1^–/–^* mice. There was a significant attenuation in the recruitment of neutrophils (4.686 ± 1.65 vs. 9.725 ± 1.44; *P* = 0.021) on day 1 and inflammatory macrophages on day 1 (11.17 ± 0.81 vs. 15.52 ± 1.16; *P* = 0.016) and day 5 (9.157 ± 1.85 vs. 17.42 ± 2.11; *P* = 0.022) in bleomycin-challenged *fpr1^–/–^* mice compared with C57BL/6 mice. In contrast, recruitment of T cells on day 5 was comparable between *fpr1^–/–^* and C57BL/6 mice (*P* = 0.54) (Figure 2, E–G). Next, we assessed the phenotype of tissue macrophages (F4/80^+^CD11b^+^) in C57BL/6 and *fpr1^–/–^* mice by quantifying the cell surface expression of iNOS (M1 macrophage marker) and CD206 (M2 macrophage marker) by flow cytometry. There was little to no difference in the total number of macrophages (*P* = 0.48) or the number of iNOS^+^ M1 macrophages (*P* = 0.43) in challenged C57BL/6 mice compared with *fpr1^–/–^* mice. The number of CD206^+^ M2 macrophages was significantly elevated on day 21 in challenged C57BL/6 mice compared with control mice. In contrast, the frequency of CD206^+^ M2 macrophages in bleomycin-challenged *fpr1^–/–^* mice was not significantly different than control mice (*P* = 0.93) and was significantly less than in C57BL/6 mice (13.22 ± 2.38 vs. 34.16 ± 6.88; *P* = 0.019) (Figure 2, H–J).

**Figure 3:**
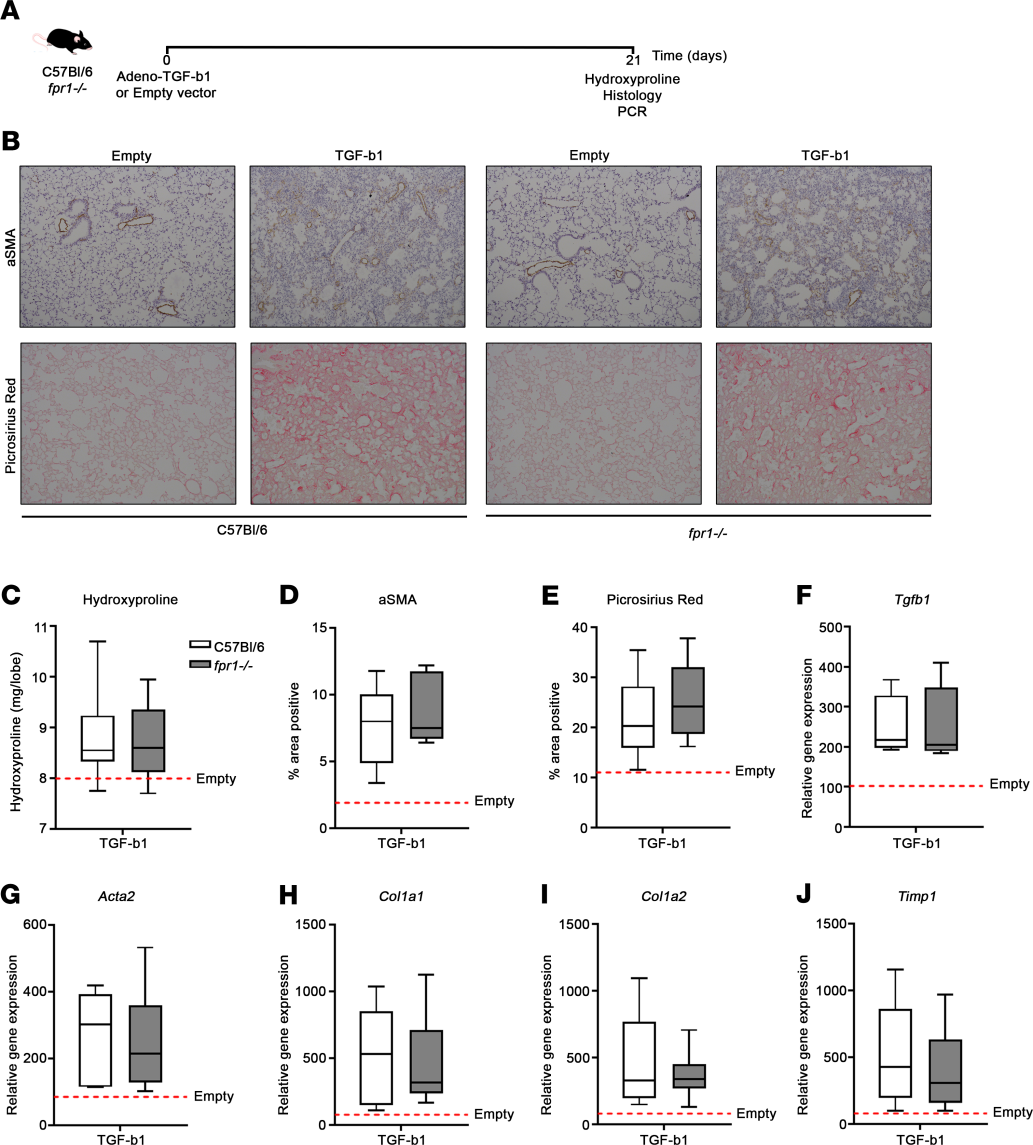
*fpr1^–/–^* mice are not protected from adeno-TGF-β1–induced lung fibrosis. (**A**) C57BL/6 and *fpr1^–/–^* mice were challenged intratracheally with 4 × 10^8^ PFUs of TGF-β1 adenovirus (Ad-TGF-β1^223/225^) or control virus (Ad-DL) in 30 μl sterile saline and lung tissue**–**harvested on day 21. (**B**) Representative α-smooth muscle actin–stained (αSMA-stained) and Picrosirius red–stained lung tissue on day 21. (**C**) Hydroxyproline (μg/lobe) content of lung tissue on days 5 and 21. (**D**) Percentage area positive of αSMA and (**E**) Picrosirius red staining on day 21. Data represent the mean value of 20 randomly selected, nonoverlapping fields (original magnification, ×20). Relative gene expression of TGF-β1 (**F**), αSMA (**G**), Collagen 1a1 (**H**), Collagen 1a2 (**I**), and TIMP1 (**J**) in lung tissue on day 21 was assessed by qPCR. Gene expression was normalized to GAPDH as a loading control. *n* = 6–8 mice per group. No significant difference was seen between control virus–treated C57BL/6 and *fpr1^–/–^* mice, and therefore control virus mice were pooled and presented as mean (red-hashed line). Data were analyzed using a Mann-Whitney *U* test and presented as box-and-whisker plots. All *P* > 0.05.

To determine whether FPR-1 was directly regulating the fibrogenic response, we exposed C57BL/6 and *fpr1^–/–^* mice to AdTGF-β1^223/225^ or adenovirus null vector control (4 × 10^8^ PFUs) and harvested on day 21 to assess fibrosis (Figure 3A). AdTGF-β1^223/225^ induced fibrosis to a similar degree in C57BL/6 and *fpr1^–/–^* mice, as indicated by an elevation in total lung collagen content (Figure 3C), an increase in the percentage area positive for Picrosirius red (Figure 3, B and E) and αSMA (Figure 3, B and D), and an increase in fibrogenic gene expression (Figure 3, F–J). These data suggest that FPR-1 is not directly involved in the fibrogenic response in the lung, but instead contributes to inflammation by driving immune cell recruitment and activation in response to tissue damage.

### FPR-1 is not required for the initiation or progression of fibrosis in the liver or kidney.

**Figure 4:**
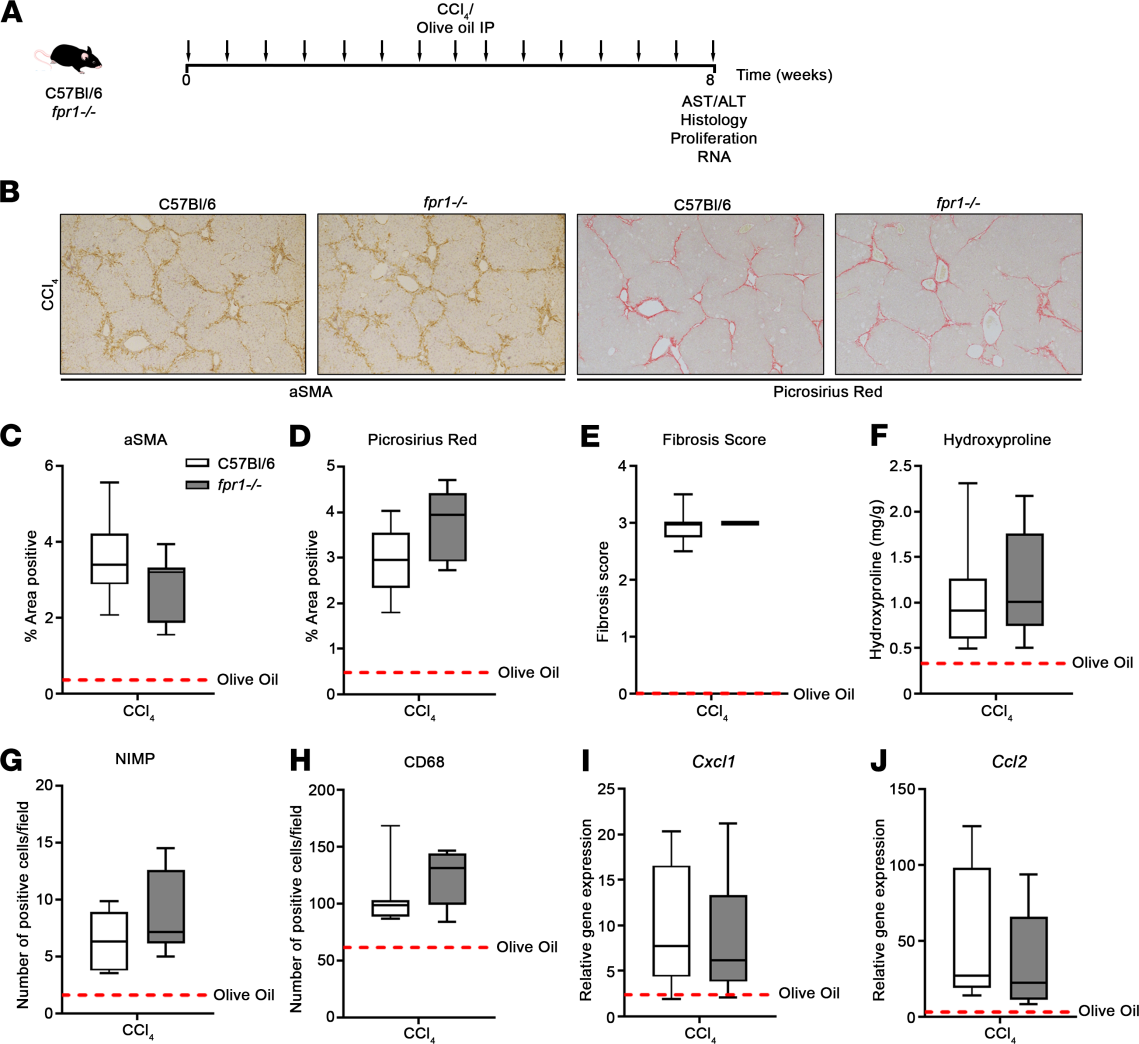
*fpr1^–/–^* mice are not protected from CCl_4_-induced liver fibrosis. (**A**) C57BL/6 and *fpr1^–/–^* mice were challenged biweekly for 8 weeks with i.p. injection of carbon tetrachloride (CCl_4_) at 2 μl/g body weight (CCl_4_/olive oil at 1:3 [vol/vol]) or olive oil as a control. Mice were killed 24 hours after the last CCl_4_ injection. (**B**) Representative α-smooth muscle actin–stained (αSMA-stained) and Picrosirius red–stained liver tissue. Percentage area positive of (**C**) αSMA and (**D**) Picrosirius red staining. Data represent the mean value of *n* = 20 randomly selected, nonoverlapping fields (original magnification, ×10). (**E**) Fibrosis score (0–4) according to the degree of collagen staining and scar formation. (**F**) Hydroxyproline (μg/g) content of liver tissue. Number of (**G**) NIMP^+^ and (**H**) CD68^+^ cells per field (original magnification, ×20). Data represent the mean value of *n* = 20 randomly selected, nonoverlapping fields per mouse. Relative gene expression of KC (**I**) and MCP1 (**J**) in liver tissue was assessed by qPCR. Gene expression was normalized to GAPDH as a loading control. *n* = 5–9 mice per group. No significant difference was seen between olive oil–treated C57BL/6 and *fpr1^–/–^* mice, and therefore olive oil–treated mice were pooled and presented as mean (red-hashed line). Data were analyzed using a Mann-Whitney *U* test and presented as box-and-whisker plots. All *P* > 0.05. ALT, alanine transaminase; AST, aspartate aminotransferase.

To investigate whether FPR-1 plays proinflammatory and/or profibrotic roles in the liver, we first utilized acute and chronic carbon tetrachloride-induced (CCl_4_-induced) models of liver injury and fibrosis. In the acute model, C57BL/6 and *fpr1^–/–^* mice were given a single i.p. injection of CCl_4_ or olive oil (as a control) and were harvested after 24, 48, or 72 hours (Supplemental Figure 1A; supplemental material available online with this article; https://doi.org/10.1172/jci.insight.125937DS1). In the chronic model, C57BL/6 and *fpr1^–/–^* mice were given biweekly IP injection of CCl_4_ or olive oil for 8 weeks and were harvested 24 hours after the last dose of CCl_4_ (Figure 4A). The degree of liver injury as quantified by serum aspartate aminotransferase (AST) and alanine transaminase (ALT) levels was comparable in both C57BL/6 and *fpr1^–/–^* mice (Supplemental Figure 2, A and B). Similarly, and in contrast to the lung, recruitment of neutrophils (NIMP^+^) and inflammatory macrophages (CD68^+^) was comparably elevated in both C57BL/6 and *fpr1^–/–^* mice after acute (Supplemental Figure 1, D and E) or chronic (Figure 4, G and H) CCl_4_ challenge. Moreover, proliferation, as quantified by the number of PCNA^+^ hepatocytes (Supplemental Figure 1F and Supplemental Figure 2C) and inflammatory gene expression (Figure 4, I and J), was comparably elevated in both C57BL/6 and *fpr1^–/–^* mice. Activation of quiescent hepatic stellate cells in response to CCl_4_ is associated with the upregulation of α-SMA expression. Both C57BL/6 and *fpr1^–/–^* mice had markedly increased numbers of αSMA^+^ myofibroblasts (Supplemental Figure 1G) 48 hours and 72 hours after CCl_4_ challenge, and this increase was maintained in chronic CCl_4_-challenged mice (Figure 4, B and C). Importantly, fibrosis, as measured histologically (Figure 4, B, D, and E) and biochemically (Figure 4F), was comparable between C57BL/6 and *fpr1^–/–^* mice, suggesting that FPR-1 is not required for initiation or progression of liver fibrosis in response to CCl_4_ challenge.

**Figure 5:**
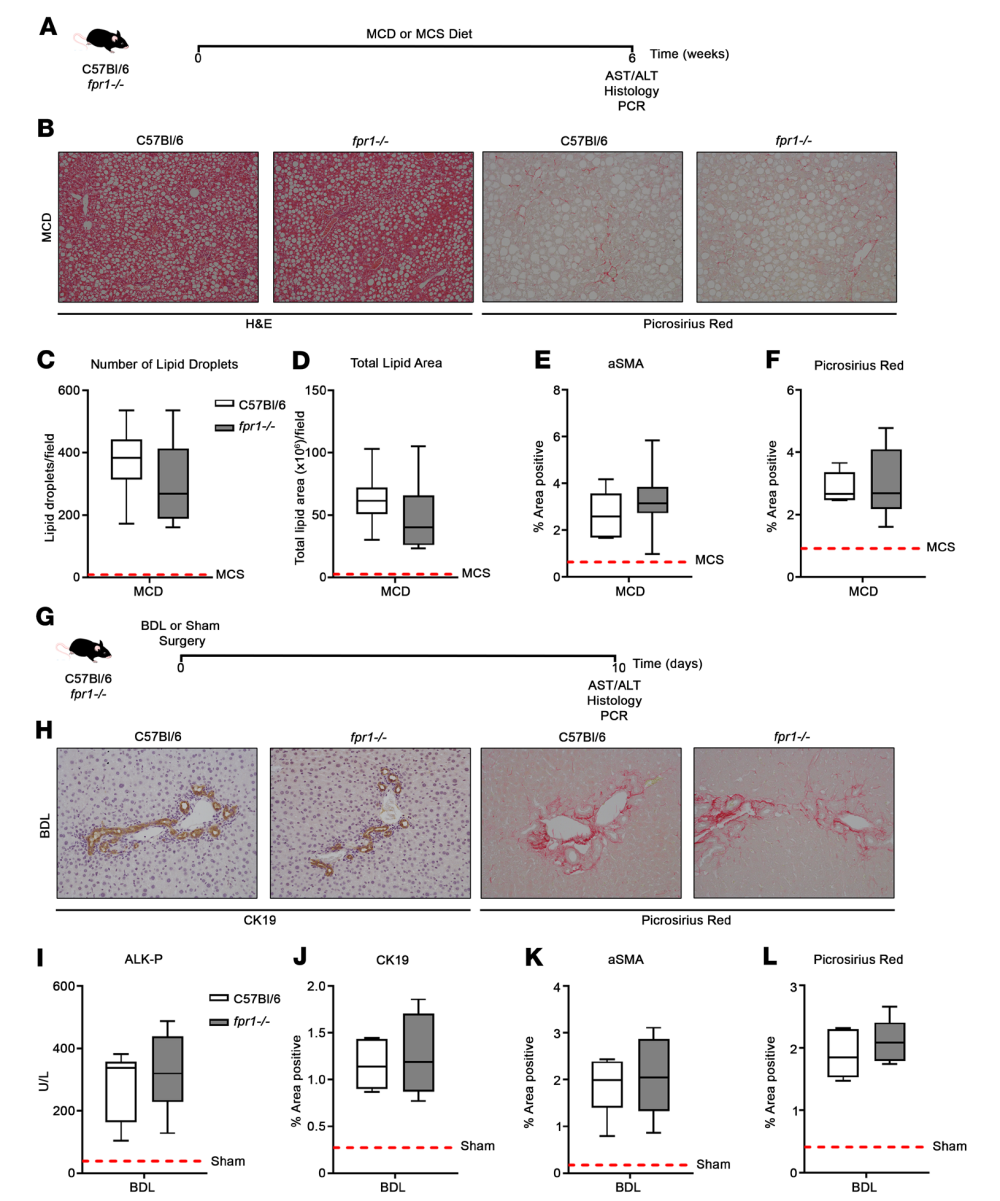
*fpr1^–/–^* mice are not protected from MCD- or BDL- induced liver fibrosis. (**A**) C57BL/6 and *fpr1^–/–^* mice were fed a methionine/choline-deficient (MCD) diet or control methionine/choline sufficient (MCS) diets and then harvested on week 6. (**B**) Representative H&E- and Picrosirius red–stained liver tissue. (**C**) The total number of lipid droplets and (**D**) total lipid area were quantified from H&E-stained liver sections. Percentage area positive of (**E**) α-smooth muscle actin (αSMA) and (**F**) Picrosirius red staining. Data represent the mean value of *n* = 20 randomly selected, nonoverlapping fields (original magnification, ×10). (**G**) C57BL/6 and *fpr1^–/–^* mice underwent bile duct ligation (BDL) and were harvested 10 days after surgery. (**H**) Representative cytokeratin-19– (CK19-) and Picrosirius red–stained liver tissue. (**I**) Serum alkaline phosphatase (ALK-P) levels expressed as units/liters (U/L) in BDL-injured mice. Percentage area positive of (**J**) CK19-, (**K**) αSMA-, and (**L**) Picrosirius red–stained liver sections. Data represent the mean value of *n* = 20 randomly selected, nonoverlapping fields (original magnification, ×10). *n* = 6–10 mice per group for **A**–**F** and *n* = 5–9 mice per group for **G**–**L**. Data were analyzed using a Mann-Whitney *U* test and presented as box-and-whisker plots. All *P* > 0.05. ALT, alanine transaminase; AST, aspartate aminotransferase; BDL, bile duct ligation; MCD, methionine/choline deficient; MCS, methionine/choline sufficient.

To conclusively evaluate the potential importance of FPR-1 in the liver, C57BL/6 and *fpr1^–/–^* mice were assessed in 2 further models of liver fibrosis driven by very different insults: the methionine/choline-deficient (MCD) diet model (Figure 5A) and the bile duct ligation (BDL) model (Figure 5G). Hepatic steatosis, as characterized by the number of lipid droplets and total lipid area (Figure 5, B–D), and fibrosis, as characterized by an increase in the percentage area positive for Picrosirius red and αSMA (Figure 5, B, E, and F, and Supplemental Figure 3A) or fibrogenic gene expression (Supplemental Figure 3C), were comparable between C57BL/6 and *fpr1^–/–^* mice in the MCD diet model. Similarly, biliary expansion, as characterized by an increase in the percentage area positive for cytokeratin-19 (CK19), in response to similar levels of liver injury (Figure 5, H–J), and periportal fibrosis, as characterized above (Figure 5, H, K, and L, and Supplemental Figure 3, D and F), were comparable between C57BL/6 and *fpr1^–/–^* mice in the BDL model. Collectively, these data demonstrate that FPR-1 is not required for the development of hepatic fibrosis.

**Figure 6:**
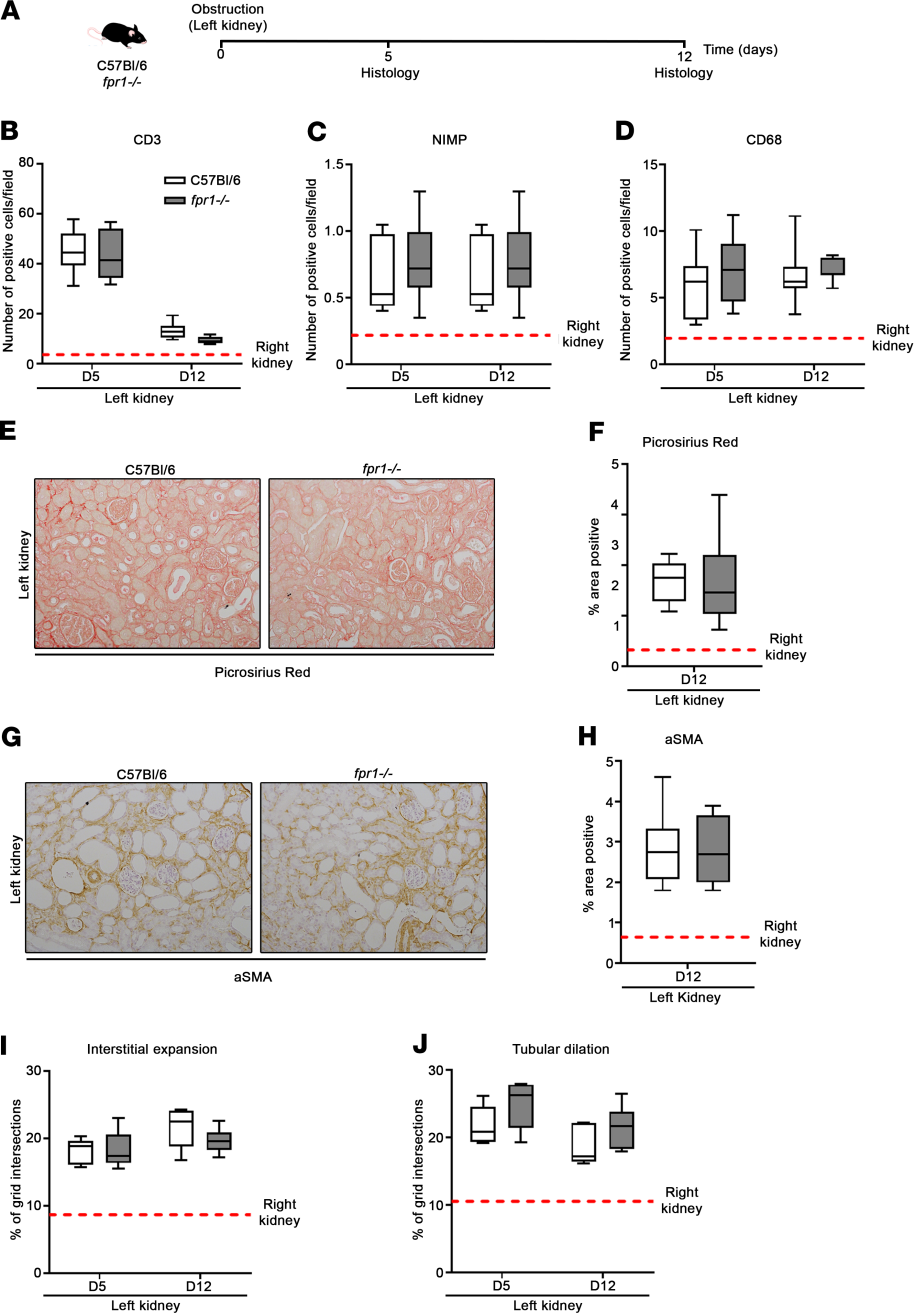
*fpr1^–/–^* mice are not protected from fibrosis in the unilateral ureteral obstruction model of kidney injury. (**A**) The left kidney of C57BL/6 and *fpr1^–/–^* mice was obstructed by ligation of the corresponding ureter. The right kidney underwent the same surgical procedure without ligation. Mice were killed on days 5 and 12 after surgery and kidney tissue harvested. Number of (**B**) CD3^+^, (**C**) NIMP^+^, and (**D**) CD68^+^ cells per field (original magnification, ×20). Data represent the mean value of *n* = 20 randomly selected, nonoverlapping fields per mouse. Representative (**E**) Picrosirius red–stained and (**G**) α-smooth muscle actin–stained (αSMA-stained) kidney tissue. Percentage area positive of (**F**) Picrosirius red and (**H**) αSMA staining. Data represent the mean value of *n* = 20 randomly selected, nonoverlapping fields (original magnification, ×10). (**I**) Interstitial expansion and (**J**) tubular dilation as a percentage of grid intersections (excluding glomeruli) overlaying interstitial areas and tubular lumine. Data represent the mean value of *n* = 20 randomly selected, nonoverlapping fields (original magnification, ×20). *n* = 6–7 mice per group. No significant difference was seen between the right kidneys of C57BL/6 and *fpr1^–/–^* mice, and therefore mice were pooled and presented as mean (red-hashed line). Data were analyzed using a Mann-Whitney *U* test and presented as box-and-whisker plots. *P* > 0.05.

To determine the role of FPR-1 in renal fibrosis, we utilized the unilateral ureteral obstruction (UUO) model of kidney injury, whereby surgical ligation of the ureter of the left kidney triggers an increase in hydrostatic pressure driving damage, inflammation, and ultimately fibrosis (Figure 6A). Immune cell recruitment, as quantified by the number of T cells (CD3^+^), neutrophils (NIMP^+^), and monocytes (CD68^+^) in tissue on day 5 and day 12 after UUO, was comparably elevated in both C57BL/6 and *fpr1^–/–^* mice (Figure 6, B–D). Moreover, fibrosis, as quantified by percentage area positive for Picrosirius red (Figure 6, E and F), and myofibroblast activation, as quantified by percentage area positive for αSMA (Figure 6, G and H), were equally elevated in both C57BL/6 and *fpr1^–/–^* mice. Finally, interstitial expansion (Figure 6I) and tubular dilatation (Figure 6J) were quantified histologically and were not significantly different between C57BL/6 and *fpr1^–/–^* mice. Taken together, these data suggest that FPR-1 is not required for initiation or progression of renal fibrosis in the UUO model.

### Defective recruitment of fpr1^–/–^ neutrophils to the lung in response to injury.

Neutrophil recruitment to the liver and kidney in response to CCl_4_ challenge or UUO-induced renal injury, respectively, was comparable between C57BL/6 and *fpr1^–/–^* mice. In contrast, neutrophil recruitment to the lungs of *fpr1^–/–^* mice in response to bleomycin-induced tissue injury was attenuated compared with C57BL/6 mice. Taken together, these data suggest that FPR-1 is required for effective homing of neutrophils to the lung but not the liver or kidney. However, whether this is an intrinsic defect in the neutrophil or a defect in chemokine gradients in the lung of *fpr1^–/–^* mice requires further investigation.

**Figure 7:**
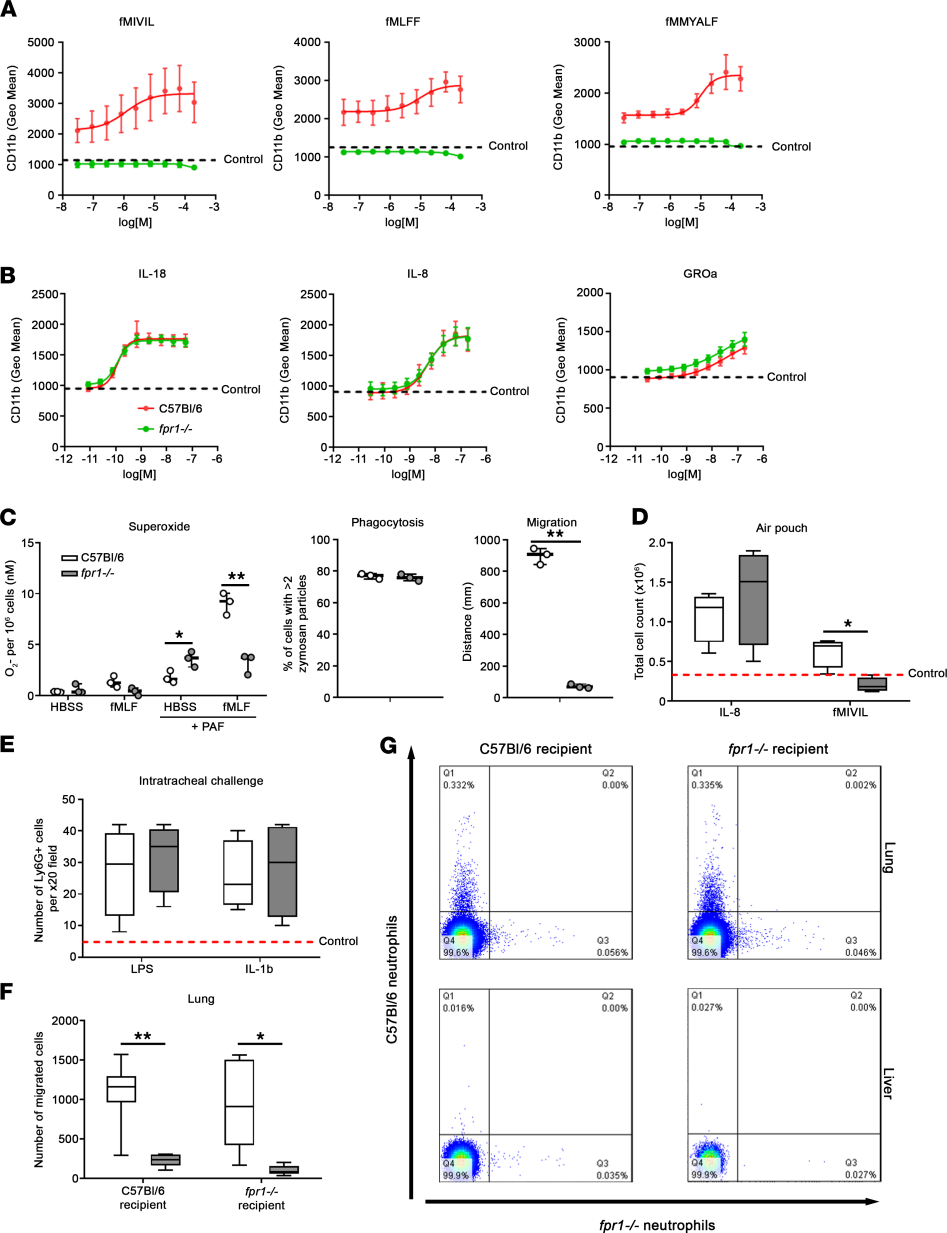
FPR-1 is required for effective neutrophil migration to injured lung tissue. Bone marrow derived cells from C57BL/6 and *fpr1^–/–^* mice were stimulated with formyl peptides (fMIVIL, fMLFF, fMMYALF) (**A**) or IL-18, IL-8, and GROα (**B**) and the change in CD11b expression on neutrophils (GR1^+^ cells) assessed by flow cytometry. (**C**) Bone marrow–derived neutrophils (>95% purity) from C57BL/6 and *fpr1^–/–^* mice were assessed for their ability to release superoxide**,** phagocytose zymosan particles and migrate toward formyl peptides. (**D**) C57BL/6 and *fpr1^–/–^* mice were stimulated intrapouch for 4 hours with IL-8 (250 μM) or fMIVIL (3 μM) and total cell recruitment measured in an inflammatory subcutaneous air pouch model by flow cytometry. Dotted line indicates mean total cell influx observed for control group with carboxymethyl cellulose carrier alone. (**E**) C57BL/6 and *fpr1^–/–^* mice were challenged intratracheally with saline (30 μl), LPS (1 μg/mL), or IL-1β (1 μg/mL) and lung tissue harvested on day 1. Data represent the mean number of Ly6G^+^ cells of *n* = 20 randomly selected, nonoverlapping fields (original magnification, ×20) per mouse. Dotted line indicates mean number of Ly6G^+^ cells observed for saline-challenged mice. Bone marrow–derived neutrophils (>95% purity) were isolated from C57BL/6 and *fpr1^–/–^* mice, labeled (CFSE, C57BL/6; Violet, *fpr1^–/–^*), mixed (1:1 ratio), and injected via a single intravenous injection into recipient (C57BL/6 and *fpr1^–/–^*) mice. After transfer of cells, mice were challenged with bleomycin to induce acute pulmonary injury and culled after 2 hours. Lung (**G**, top) and liver homogenates (**G**, bottom) were analyzed by flow cytometry to quantify neutrophil migration (**F** and **G**). *n* = 6–7 mice per group for **A**–**E** and *n* = 8 mice per group for **F** and **G**. Data were analyzed using a Mann-Whitney *U* test and presented as box-and-whisker plots. *P < 0.05; **P < 0.01.

The inability of *fpr1^–/–^* neutrophils to activate in response to challenge with formyl peptides (fMIVIL, fMLFF, fMMYALF) was confirmed by a failure to upregulate CD11b expression compared with neutrophils isolated from C57BL/6 mice (Figure 7A). In contrast, neutrophils isolated from both *fpr1^–/–^* and C57BL/6 mice were activated by IL-18, IL-8, and GROα at comparable doses and to a comparable degree, ruling out heterologous desensitization of common chemotactic receptors on loss of FPR-1 (Figure 7B). Moreover, while phagocytosis of zymosan particles was comparable between neutrophils isolated from both *fpr1^–/–^* and C57BL/6 mice, the production of superoxide in response to challenge with formyl peptides and the ability to migrate toward formyl peptides was attenuated in *fpr1^–/–^* neutrophils (Figure 7C). An air pouch model in C57BL/6 and *fpr1^–/–^* mice was used to further investigate neutrophil recruitment in vivo. Critically, the number of neutrophils recruited to the air pouch in response to IL-8 was comparable between C57BL/6 and *fpr1^–/–^* mice, whereas neutrophil recruitment was defective in *fpr1^–/–^* mice when responding to formyl peptides (Figure 7D). Comparable levels of activation (as measured by upregulation of CD11b and CD66b on GR1^+^ cells) of neutrophils recruited in response to IL-8 was observed between C57BL/6 and *fpr1^–/–^* mice; however, neutrophils were not recruited/activated in response to formyl peptide (fMIVIL) in *fpr1^–/–^* mice (data not shown).

We next investigated neutrophil recruitment to the lungs of C57BL/6 and *fpr1^–/–^* mice in response to challenge with LPS or IL-1β (both 1 μg/mL) to determine whether impaired neutrophil recruitment in *fpr1^–/–^* mice was specific to lung injury induced by bleomycin or was seen in response to other inflammatory challenges. Critically, neutrophil numbers in the lungs were comparably elevated 1 day after LPS or IL-1β challenge in C57BL/6 and *fpr1^–/–^* mice (Figure 7E), thus confirming that FPR-1–deficient neutrophils are effectively recruited in response to non-FPR-1 ligands. Taken together, these data show that *fpr1^–/–^* neutrophils can be activated by and recruited toward a number of chemokines commonly elevated in damaged and fibrotic lung tissue, being exclusively defective in their ability to respond to FPR-1 ligands.

Next, we performed an adoptive transfer experiment in C57BL/6 and *fpr1^–/–^* mice to determine whether *fpr1^–/–^* neutrophils could be effectively recruited to the lungs of injured C57BL/6 mice. To do this, we isolated neutrophils from C57BL/6 and *fpr1^–/–^* donor mice and labeled them ex vivo with CFSE (C57BL/6) or Violet (*fpr1^–/–^*). Labeled cells were mixed 1:1 and injected intravenously (1.5 × 10^6^ cells) into bleomycin-injured recipient mice (C57BL/6 and *fpr1^–/–^*). After 2 hours, mice were culled and lung and liver homogenates were analyzed by flow cytometry to quantify neutrophil migration. CFSE-labeled C57BL/6 neutrophils were effectively recruited to the injured lung in both C57BL/6 and *fpr1^–/–^* mice, suggesting that the chemokine gradients in the lungs of *fpr1^–/–^* mice can effectively recruit neutrophils. In contrast, the number of Violet-labeled *fpr1^–/–^* neutrophils recruited was significantly attenuated in both C57BL/6 and *fpr1^–/–^* mice, suggesting that the defect lies with the *fpr1^–/–^* neutrophils rather than the lung microenvironment (Figure 7, F and G). Little to no nonspecific recruitment of labeled neutrophils was observed to the uninjured liver (Figure 7G). These data suggest that FPR-1 expression on neutrophils is required for their effective homing to the lung after injury, and by extension, FPR-1 ligands are the primary neutrophil recruitment signal produced by the bleomycin-challenged lung.

### Neutrophil depletion reduces bleomycin-induced lung inflammation and fibrosis.

**Figure 8:**
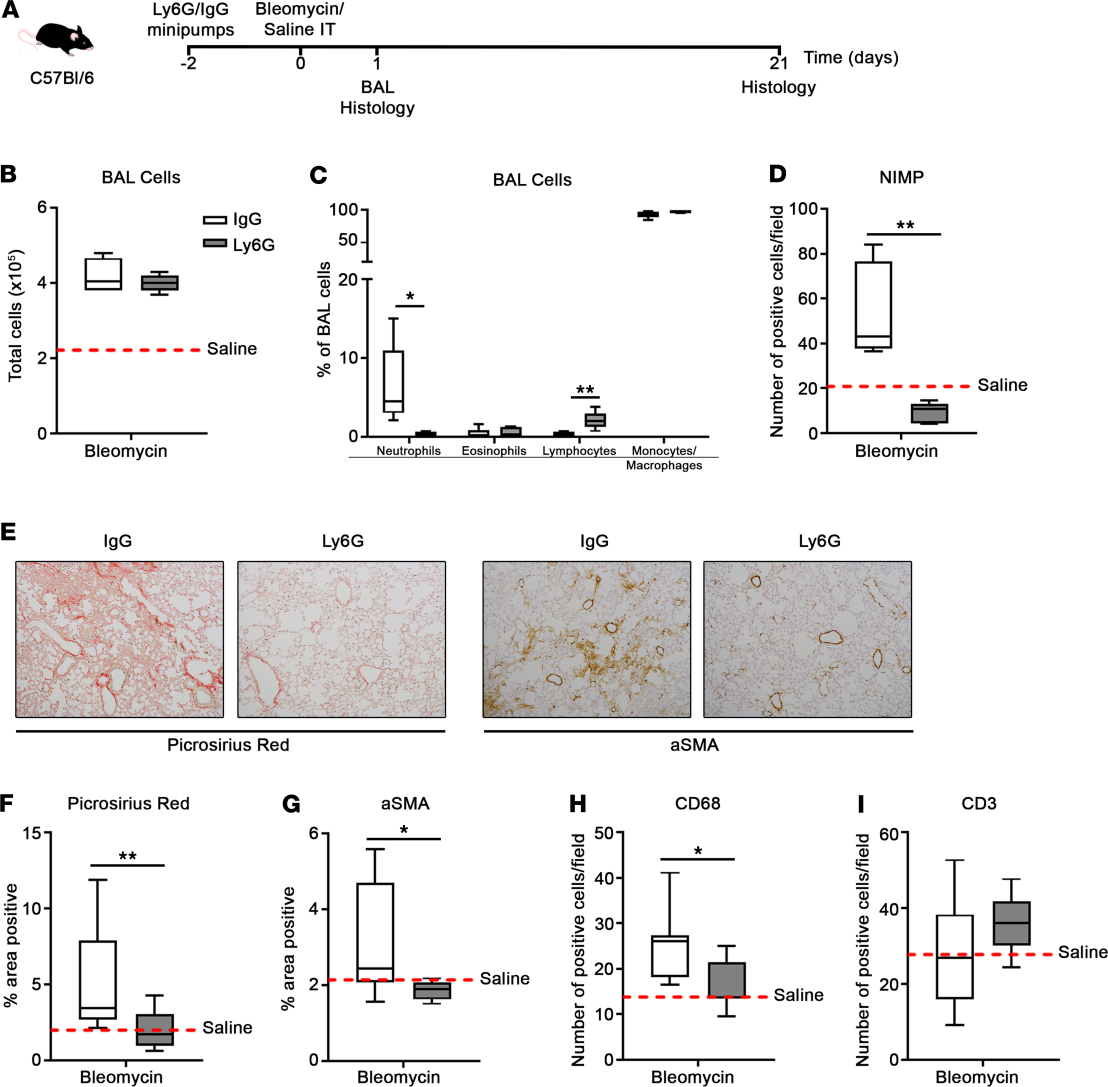
Neutrophil depletion attenuates bleomycin-induced lung fibrosis. (**A**) Osmotic minipumps loaded with anti-Ly6G or IgG2a control antibodies were implanted subcutaneously into C57BL/6 mice. After 48 hours the mice were challenged intratracheally with saline (30 μl) or bleomycin sulfate (0.007 U in 30 μl saline) and bronchoalveolar lavage (BAL) and lung tissue**–**harvested on days 1 and 21. Total cell numbers (**B**) and percentage of neutrophils, eosinophils, lymphocytes, and monocytes/macrophages (**C**) in BAL on day 1 were assessed by cytospin differential cell counts. (**D**) Number of NIMP^+^ cells per field (original magnification, ×20) on day 1. Data represent the mean value of *n* = 20 randomly selected, nonoverlapping fields per mouse. (**E**) Representative Picrosirius red– and α-smooth muscle actin–stained (αSMA-stained) lung tissue on day 21. Percentage area positive for (**F**) Picrosirius red and (**G**) αSMA staining on day 21. Data represent the mean value of *n* = 20 randomly selected, nonoverlapping fields (original magnification, ×10). Number of (**H**) CD68^+^ and (**I**) CD3^+^ cells per field (original magnification, ×20) on day 21. Data represent the mean value of *n* = 20 randomly selected, nonoverlapping fields per mouse. No significant difference was seen between saline-treated C57BL/6 and *fpr1^–/–^* mice, and therefore saline-treated mice were pooled and presented as mean (red-hashed line). *n* = 5 mice per group for **A**–**D** and *n* = 8–9 mice per group for **E**–**I**. Data were analyzed using a Mann-Whitney *U* test and presented as box-and-whisker plots. *P < 0.05; **P < 0.01. IT, intratracheal.

We next determined whether neutrophils are required for lung inflammation and fibrosis by carrying out a neutrophil depletion experiment. To deplete neutrophils, we surgically implanted minipumps containing either a Ly6G 1A8–depleting antibody (20) or an IgG isotype control antibody 2 days before bleomycin challenge. We assessed the efficacy of neutrophil depletion on day 1 and quantified inflammation and fibrosis on day 21 (Figure 8A). On day 1, the total number of cells in the bronchoalveolar lavage (BAL) was not significantly different between bleomycin-challenged IgG- and Ly6G-treated mice, with both elevated above saline-challenged mice (Figure 8B). However, neutrophils as a percentage of BAL cells (Figure 8C) and the number of neutrophils (NIMP^+^) in lung tissue, as assessed by IHC, were significantly reduced in Ly6G-treated mice (Figure 8D). Critically, fibrosis, as quantified by Picrosirius red (Figure 8, E and F) and αSMA (Figure 8, E and G), was significantly attenuated in Ly6G-treated mice compared with IgG-treated mice, with levels of both comparable to saline-challenged mice. Similarly, the number of inflammatory macrophages (CD68^+^) in lung tissue was attenuated in Ly6G-treated mice but the number of T cells (CD3^+^) was not affected (Figure 8, H and I). These data suggest that neutrophils are required for efficient recruitment of inflammatory macrophages to the damaged lung and for the development of fibrosis. Moreover, FPR-1 and its ligands are critical for regulating neutrophil recruitment, and, as such, they are likely to be critical molecular initiators of the cellular events that lead to fibrosis and should be considered further as novel antifibrotic therapies in the lung.

## Discussion

Fibrosis is the dysregulated accumulation of collagen and ECM constituents and can affect any tissue or organ (3). The mechanisms underlying fibrosis can be either core, involved in the initiation or progression of fibrosis in multiple organs, or organ specific. In this study, we describe a tissue-specific role for FPR-1 in the development of pulmonary fibrosis, with FPR-1 being entirely dispensable in the development of hepatic and renal fibrosis. Mechanistically, we show that FPR-1 is required for effective homing of neutrophils to damaged lung tissue and that depletion of neutrophils protects mice from bleomycin-induced pulmonary fibrosis. A limitation of this study is that conclusions have been drawn exclusively from murine models of fibrosis, predominantly the bleomycin-induced lung fibrosis model. Although this model of lung fibrosis can be criticized (it is performed in young animals [8–10 weeks], with rapid onset of disease [21 days] and is induced by a nonphysiologically relevant stimulus), it is still regarded as the reluctant gold standard for the field and provides the foundation for the majority of preclinical studies performed (21–23). Another criticism of the model is whether bleomycin-induced acute lung injury resolves in those animals that survive. Recently, Limjunyawong et al. followed mice for 6 months after a single bleomycin challenge and found that while there is some repair, the injury never fully resolves, with persistent fibrosis that may have similarities to many features of human idiopathic pulmonary fibrosis (IPF) (24).

Neutrophils are the most abundant inflammatory cells at the earliest stages of wound healing but are subsequently replaced by infiltrating inflammatory macrophages after neutrophil degranulation. We observed that depletion of neutrophils prevents inflammatory macrophage recruitment to the damaged lung, indicating an important initiator role for neutrophils in lung inflammation that is required for subsequent inflammatory macrophage-driven events. Of note, it was recently reported in a model of sterile liver injury that recruited neutrophils eventually leave the organ by a highly regulated process and that this migration back into the peripheral circulation is a requirement for subsequent monocyte recruitment (25). Our data therefore support emerging evidence that neutrophils are important regulators of inflammatory macrophage infiltration to damaged tissues. Despite their relatively short temporal presence, neutrophils play a critical role in preventing infection through the generation of antimicrobial peptides, reactive oxygen species, and proteases. However these mediators may also damage the host and can have long-lasting effects on the speed and quality of tissue repair (26). Recently, there have been a number of reports that suggest neutrophils play an important role in the initiation and progression of pulmonary fibrosis. For example the depletion of neutrophils using anti-GR1 or anti-Ly6G antibodies significantly decreased pulmonary inflammation and fibrosis in murine models of hypersensitivity pneumonitis (27) and *Paracoccidioides brasiliensis*–induced pulmonary fibrosis (28). Mice deficient in P2Y2 fail to recruit neutrophil and have attenuated fibrosis in response to bleomycin challenge and treatment of mice with a CXCR2 antagonist reduced airway neutrophil transmigration and pulmonary fibrosis (29, 30). Moreover, a number of studies have demonstrated a critical role for neutrophil elastase in pulmonary fibrosis. Neutrophil elastase–null mice are protected from bleomycin-induced pulmonary fibrosis (31) and a similar protective effect is seen in mice treated with the neutrophil elastase inhibitor sivelestat (32). Critically, the number of neutrophils and levels of the neutrophil chemoattractant IL-8 are increased in patients with IPF compared with healthy subjects (33), and neutrophilia has been shown to predict early mortality in patients with IPF (34). Taken together, these data imply a critical role for neutrophils and their secreted products in the initiation and progression of fibrosis in the lung. Here, we provide strong experimental evidence that FPR-1 is essential for participation of neutrophils in lung fibrosis and suggest that the receptor and/or its ligands are rational therapeutic targets.

In contrast, the role of neutrophils in hepatic and renal fibrosis is less clear, with some studies reporting little to no involvement and others showing a critical role for neutrophils (35–38). Recently, it has been reported that infusion of neutrophils into mice with established CCl_4_-induced liver fibrosis significantly reduced collagen accumulation, suggesting that neutrophils can effectively reduce fibrosis. The same study also demonstrated that depletion of neutrophils significantly delayed resolution of liver fibrosis, indicating that neutrophils are involved in natural fibrosis resolution in the liver (39). Overall, these data and our own support the idea that neutrophils play a pathological role in the development and progression of pulmonary fibrosis but add little to the uncertainty regarding the role of neutrophils in the development of hepatic and renal fibrosis.

An important role for formyl peptides and FPR-1 in the lung has previously been described (40–42). Acute respiratory distress syndrome (ARDS) is a neutrophil-dominant lung disease and formyl peptides are found at elevated levels in BAL fluid and serum from patients with ARDS. Intratracheal installation of hydrochloric acid (pH 2.0) acts as a model of sterile lung injury in mice and demonstrated a critical role for FPR-1 in recruitment of neutrophils and inflammation (40). Studies have also demonstrated that neutrophils are increased in chronic obstructive pulmonary disease (COPD) lungs and that neutrophil-associated products correlate with the development and severity of COPD (41). The peptide fMLF is an active component of cigarette smoke, a primary risk factor for the development of COPD. Compared with WT mice, *fpr1^–/–^* mice displayed a decrease in the migration of neutrophils to the lung after exposure to cigarette smoke (42).

The ability of bacterial and mitochondrial formyl peptides to act as neutrophil chemoattractants is well established; however, neutrophils are also uniquely sensitive to a vast array of other chemoattractants, including complement fragments, lipid mediators, and a multitude of chemokines (43). Our data strongly argue that recruitment of neutrophils to the lungs of injured mice occurs primarily through ligands that signal via FPR-1, as non-FPR-1 ligands (IL-1β and LPS) effectively recruited *fpr1^–/–^* neutrophils to the lungs. However, whether FPR-1–mediated recruitment is via binding of FPR-1 to mitochondrial formyl peptides (8, 9) or one of the other recently identified alternative ligands (10–12) is not clear. Moreover, the human FPR family has 3 members, namely, FPR-1, FPR-2, FPR-3, whereas the mouse FPR family is more complex and contains at least 8 related genes, 3 of which are expressed on immune cells and have similarities to human FPR-1, FPR-2, and FPR-3 (14, 44–46). The failure to recruit *fpr1^–/–^* neutrophils to the lung in response to injury indicates that the other members of the FPR family are unable to compensate for the loss of FPR-1. In contrast, *fpr1^–/–^* neutrophils are effectively recruited to the liver and kidney in response to injury, suggesting that other receptors or potentially other members of the FPR family are the primary drivers of neutrophil recruitment in these organs.

In summary, we have shown that FPR-1 expression on neutrophils is required for effective homing of neutrophils to the damaged lung. Moreover, we show that *fpr1^–/–^* mice and neutrophil-depleted C57BL/6 WT mice are protected from bleomycin-induced pulmonary fibrosis, highlighting the potential therapeutic benefit of targeting FPR-1 or neutrophils in the fibrotic lung.

## Methods

### Lung inflammation and fibrosis.

C57BL/6 and fpr1^–/–^ mice (male and female, 8–10 weeks old) were anesthetized with an i.p. injection of ketamine (80 mg/kg)/xylazine (8 mg/kg) before intratracheal challenge with a single dose of saline (30 μl), bleomycin sulfate (0.007 U in 30 μl saline), IL-1β (1 μg/mL in 30 μl saline), or LPS ([MilliporeSigma] 1 μg/mL in 30 μl saline). Mice were killed by sodium pentobarbital overdose 1, 5, or 21 days after challenge. BAL was performed by cannulating the trachea with an Insyte venous catheter (BD Bioscience) and administering ice-cooled PBS (1 mL) supplemented with EDTA (5 mM). Cells were isolated for total (EVE cell counter [NanoEntek]) and differential cell counts (Wrights-Giemsa) with the supernatant stored for analysis. Matching lung lobes were harvested for RNA isolation, protein isolation, histology, and hydroxyproline assay.

### TGF-β1 adenovirus-induced lung fibrosis.

To induce lung fibrosis, C57BL/6 and *fpr1^–/–^* mice (male, 8–10 weeks old) were anesthetized before intratracheal instillation of 4 × 10^8^ PFUs of TGF-β1 adenovirus (Ad-TGF-β1^223/225^) or control virus (Ad-DL) in 30 μl sterile saline (47). This virus expresses active TGF-β1 in the lung and produces extensive and progressive fibrosis. Mice were killed by sodium pentobarbital overdose 21 days after instillation and matching lung lobes were harvested for RNA isolation, protein isolation, histology, and hydroxyproline assay.

### CCl_4_-induced liver injury and fibrosis.

To induce acute liver injury, C57BL/6 and *fpr1^–/–^* mice (male, 8–10 weeks old) were challenged with a single i.p. dose of CCl_4_ at 2 μl/g body weight (CCl_4_/olive oil at 1:1 [vol/vol]). Mice were humanely killed 24, 48, and 72 hours after challenge. To induce liver fibrosis, C57BL/6 and *fpr1^–/–^* mice (male, 8–10 weeks old) were challenged biweekly for 8 weeks with i.p. injection of CCl_4_ at 2 μl/g body weight (CCl_4_/olive oil at 1:3 [vol/vol]). Mice were killed 24 hours after the last CCl_4_ injection. For both models, several tissue samples from throughout the large lobe were pooled for RNA and protein isolation. Tissue samples from the median, right, and left large lobes were sampled for histology. ALT and AST levels in serum were measured by NHS Biochemistry, Royal Victoria Infirmary, Newcastle upon Tyne using a standard protocol.

### BDL model.

All surgical procedures were carried out according to aseptic protocol approved by the Comparative Biology Centre, Newcastle University and Animal Scientific guidelines implemented by the United Kingdom Home Office. To induce cholestatic liver disease and periportal fibrosis, C57BL/6 and *fpr1^–/–^* mice (male, 12–14 weeks old) underwent BDL. Briefly, mice were anesthetized with isoflurane; the bile duct was then exposed by ventral laparotomy and double ligated using 5-0 nylon sterile suture wire. The incision site was sealed by suturing and glue. Sham mice underwent the same procedure without ligation of the bile duct. Mice were killed 10 days after surgery and tissues were harvested for further analysis.

### MCD diet model.

To induce fibrosis on the background of hepatic steatosis and inflammation, C57BL/6 and *fpr1^–/–^* mice (male, 8–10 weeks old) were fed a MCD diet. Control mice received a methionine/choline sufficient diet. Mice were killed on week 6 and tissues were harvested for further analysis. Alkaline phosphatase (ALK-P) levels in serum were measured by NHS Biochemistry, Royal Victoria Infirmary, Newcastle upon Tyne, using a standard protocol.

### UUO-induced kidney fibrosis.

To induce kidney fibrosis, C57BL/6 and *fpr1^–/–^* mice (female, 6–7 weeks old) underwent UUO. Mice were anesthetized by isoflurane plus oxygen; the left ureter was then exposed via a ventral laparotomy and the left kidney obstructed by ligation of the corresponding ureter with 5-0 nylon sterile suture wire. The incision site was sealed by suturing and using glue. The contralateral kidney underwent the same surgical procedure without ligation and was used as a sham surgery control. Mice were killed 5 and 12 days after surgery. Tissue from the experimental (left) and control (right) kidneys were sampled for histology.

### Ly6G-mediated neutrophil depletion.

C57BL/6 and *fpr1^–/–^* mice (male, 8–10 weeks old) were anesthetized by isoflurane plus oxygen. ALZET osmotic minipumps loaded with either anti-Ly6G (2A3) or IgG2a control (1A8) antibodies (BioXcell) were implanted subcutaneously to allow systemic and continuous delivery of the antibodies. ALZET 4-week pumps (model 2004) with a release rate of 0.25 μl/hour (28.5 μg/mouse/d) were used for the 21-day time point. ALZET 7-day pumps (model 1007D) with a release rate of 0.5 μl/hour (57 μg/mouse/d) were used for the 1-day time point. Mice were challenged with a single dose of saline (30 μl) or bleomycin sulfate (0.007 U in 30 μl saline) 48 hours after implant of the minipumps, killed by sodium pentobarbital overdose on day 1 or 21, and harvested as described above.

### Air pouch inflammation model.

An air pouch was induced on the back of C57BL/6 and *fpr1^–/–^* mice (female, 6–8 weeks old) by subcutaneously injecting 2.5 mL of sterile filtered air under light isoflurane anesthesia, allowing recovery and then reinflating in the same location 72 hours later. After another 72 hours, mice were injected intrapouch with human recombinant IL-8 (250 μM) or fMIVIL (3 μM) (chosen due to its higher potency in mice) in a carboxymethyl cellulose (CMC) carrier (0.75% CMC, 0.1% DMSO in 0.5 mL PBS) to retain the protein/peptide in the pouch. CMC carrier injected intrapouch was used as a control. Mice were killed by sodium pentobarbital overdose 4 hours after challenge and the pouch lavaged with 1 mL ice-cold heparinized PBS (5 U/mL). Total cell counts and activation markers (MFI or percent of positive cells for CD11b or CD66a expression) on GR1^+^ neutrophils were assessed using flow cytometry and normalized for volume of lavage recovered.

### Primary mouse bone marrow neutrophil isolation.

Bone marrow–derived neutrophils were prepared by centrifugation of a single Percoll layer gradient as previously described (48). Neutrophils from C57BL/6 and *fpr1^–/–^* mice were evaluated in terms of their chemotactic ability, release of superoxide anions, and phagocytosis.

### Neutrophil chemotaxis.

Freshly isolated mouse bone marrow derived neutrophils (2.5 × 10^7^ cells/mL) in 1% autologous serum were added to the central wells in an agarose (2%)/gelatin (2%)/BSA (2.5%)/medium slide. Media containing fMLF (100 nm) or medium alone were added in duplicate to each slide and the slide incubated at 37°C/5% CO_2_ for 2 hours before fixation in 2.5% paraformaldehyde. Slides were stained with Giemsa solution (MilliporeSigma) and migrated cells quantified.

### ROS production.

To measure superoxide anion release, freshly isolated mouse neutrophils (1 × 10^7^ cells/mL) were resuspended in HBSS with a priming agonist (PAF 100 nm) for 10 minutes at 37°C. Subsequent stimulation with superoxide dismutase (SOD, Sigma-Aldrich, 200 U), cytochrome *c* (1 mg/mL; Sigma-Aldrich), and fMLF/HBSS (100 nm, Sigma-Aldrich) was performed for 15 minutes in a shaking water bath at 37°C. The reaction was stopped by placing the cells on ice. Supernatants were subsequently analyzed in 96-well plate at 550 nm using a plate reader with the generation of O_2_^–^ determined by the amount of superoxide dismutase–inhibitable reduction of cytochrome *c*. Results are expressed as nanomoles of superoxide anions generated per 10^6^ neutrophils, calculated using the extinction coefficient 21 × 10^3^ M^–1^cm^–1^.

### Phagocytosis.

Freshly isolated mouse neutrophils (1 × 10^6^/mL) were plated on a 24-well plate for 1 hour to allow cell adherence. Opsonized zymosan was added to each well and incubated for 1 hour, at which point excess zymosan was washed off. Cells were fixed with methanol and stained with Giemsa solution (MilliporeSigma). Phagocytosis was quantified by counting neutrophils with 2 or more engulfed zymosan particles in four randomly selected fields with a minimum 100 neutrophils per field.

### Adoptive transfer and neutrophil tracking.

Donor mice (C57BL/6 and *fpr1^–/–^*) were culled and bone marrow–derived neutrophils isolated. To enable tracking via flow cytometry, cells were fluorescently labeled with CFSE (C57BL/6) or Violet (*fpr1^–/–^*) mixed in a 1:1 ratio and injected via a single intravenous injection (1.5 × 10^6^ cells) into recipient mice (C57BL/6 and *fpr1^–/–^*). Recipient mice were intratracheally challenged with bleomycin sulfate (0.007 U in 30 μl saline) 24 hours before injection of cells to induce acute pulmonary injury and trigger neutrophil homing to the affected area. After 2 hours, mice were culled and lung/liver homogenates were analyzed by flow cytometry to track neutrophil migration.

### Fibrosis scoring of Picrosirius red–stained liver tissue.

Paraffin-embedded formalin-fixed sections (5-μm thick) were stained with 0.1% Sirius red F3B following a standard protocol. Fibrosis was scored (0–4) according to the degree of collagen staining and scar formation as follows; 0, no fibrosis; 1, mild; 2, moderate; 3, severe (>2 vessels linked and multilayered deposition); and 4, cirrhosis (multiple instances of bridging fibrosis and nodule formation).

### Scoring of interstitial expansion and tubular dilation in kidney tissue.

Paraffin-embedded formalin-fixed sections (5-μm thick) were stained with periodic acid–Schiff (PAS) following a standard protocol. Interstitial expansion and tubular dilation in the experimental and contralateral kidneys were calculated as a percentage of grid intersections (excluding glomeruli) overlaying interstitial areas and tubular lumine. A total of 20 randomly selected, nonoverlapping fields in the cortical region were scored (original magnification, ×20).

### Hydroxyproline assay.

For assessment of fibrosis, matched lung lobes were weighed and digested with 2 mL of 6N HCl to measure the quantity of hydroxyproline as previously described (49).

### IHC.

IHC was performed on paraffin-embedded formalin-fixed tissue sections (5-μm thick). Briefly, deparaffinized sections were incubated in hydrogen peroxide/methanol followed by either proteinase K (20 μg/mL) or citrate antigen retrieval. Endogenous avidin and biotin were blocked using the Vector Avidin/Biotin Blocking Kit (Vector Laboratories), and further blocking was achieved using 20% swine serum. Sections were incubated overnight with primary antibodies (αSMA [1A4, Sigma-Aldrich], NIMP [R-14, Abcam], Ly6G [1A8, BioXcell], CD68 [OABB00472, Aviva Biosystems], CD3 [CD3-12, Bio-Rad], PCNA [PC10, Abcam], and CK19 [ab84632, Abcam]). On the next day, sections were washed and incubated with biotinylated secondary antibodies, followed by VECTASTAIN Elite ABC Reagent (Vector Laboratories). Antigens were visualized using diaminobenzidine peroxidase or ALK-P and counterstained with Mayer’s hematoxylin. The number of positive cells per field was calculated by taking the mean of 20 randomly selected, nonoverlapping fields (original magnification, ×20). To calculate percentage area positive, a threshold for positive staining was defined and applied to 20 randomly selected, nonoverlapping fields (original magnification, ×10) using Nikon Elements Software.

### RNA isolation and real-time PCR.

Total RNA was isolated using the RNeasy Kit (QIAGEN) and RNA concentration was quantified by Nanodrop. Complementary DNA synthesis was performed using a Promega kit (Promega) and real-time PCR was performed with SYBR Green JumpStart Taq ready mix following the manufacturer’s instructions and normalized to GAPDH. Primer sequences used are reported in Supplemental Table 1.

### Multiplex electrochemiluminescence assay.

IL-1β, MCP1, KC, and IL-6 concentrations in lung homogenates were measured using Meso Scale Discovery electrochemiluminescence detection kits according to the manufacturer’s instructions.

### Flow cytometry analysis.

Lung leukocytes were isolated by digesting matched lung lobes in DNase (MilliporeSigma) (25 μg/mL) and Liberase (MilliporeSigma) (18 μg/mL) for 60 minutes with rocking at 37°C, crushing through 70-μm cell strainers, and centrifuging at 350 *g* for 10 minutes. Erythrocytes were eliminated using ACK Lysing Buffer (Gibco). Lung leukocyte numbers were determined by flow rate. To assess cell populations and macrophage phenotype, lung leukocytes (4 × 10^5^) were labeled with a viability dye (Invitrogen Live/Dead Aqua), preincubated with unlabeled anti-CD16/32 antibody, and stained with: CD45-APC-Cy7 (30-F11, BioLegend), CD11b-Percp (M1/70, BioLegend), F4/80-BV421 (BM8, BioLegend), CD206-APC (C068C2, BioLegend), and iNOS (K13-A, Biorbyt). Macrophages were gated as CD45^+^F4/80^+^/CD11b^+^ and then separated into distinct M1 (CD45^+^F4/80^+^/CD11b^+^/iNOS^+^) and M2 (CD45^+^F4/80^+^/CD11b^+^/CD206^+^) subsets. Flow cytometry was performed using a FACSCanto II and data were analyzed using FlowJo (Tree Star).

### Bone marrow GR1^+^ cell activation.

The method for isolating bone marrow cells from mice was derived from Southgate et al. (50). Briefly, bone marrow cells were isolated from the femurs of *n* = 3 C57BL/6 or *fpr1^–/–^* mice, pooled, and passed through a 40-μm cell strainer. Cells were resuspended in RPMI 1640 Glutamax with 2% bovine serum albumin and seeded into a 96-well, round-bottom plate at 8 × 10^4^ cells/well in a final volume of 200 μl. Cells were stimulated with a titration of human recombinant IL-8, murine recombinant IL-18, human recombinant GROα, or various formyl peptides (R&D Systems, MBL, R&D Systems and custom synthesized by CRB or Bachem, respectively). Plates were incubated at 37^o^C, 5% CO_2_ for 50 minutes and stained with anti–GR1-APC and anti–CD11b-PE to allow quantification of CD11b expression on live GR1^+^ve cells.

### Statistics.

Data were analyzed using Mann-Whitney U test. Significance was defined by a *P* value less than 0.05. Results are presented as box-and-whisker plots or individual values as appropriate.

### Study approval.

All animal experiments were conducted in accordance with the United Kingdom Animals Scientific Procedures Act of 1986, and under approval from the Newcastle Ethical Review Committee and a United Kingdom Home Office license. C57BL/6 mice were obtained from an in-house breeding colony (Comparative Biology Centre). *fpr1^–/–^* mice were originally obtained from MedImmune and subsequently maintained on a C57BL/6 background at Newcastle University.

## Author contributions

BJMM and JL performed the majority of experimental work. ADCP, BSB, RB, SL, JS, AC, JDF, JF, MAS, DR, RH, CF, and FO performed and evaluated specific experiments. AJS and JG provided materials. LAB, LAM, DKF, AJF, and DAM designed and supervised the study. LAB wrote the manuscript with contributions from LAM, DKF, AJF, and DAM.

## Supplementary Material

Supplemental data

## Version 1 02/27/2020

Electronic publication

## Version 2 03/11/2020

The copyright license was updated.

## Version 3 03/27/2020

The PDF was added.
