# Supplementary material for: FPR-1 is an important regulator of neutrophil recruitment and a tissue-specific driver of pulmonary fibrosis
Source: JCI Insight. 2020 Feb 27;5(4):e125937. doi: 10.1172/jci.insight.125937 (PMC7101152; doi:10.1172/jci.insight.125937)
Supplement: Supplemental data [file jciinsight-5-125937-s272.pdf]

FPR-1 is an important regulator of neutrophil recruitment and a tissue-specific driver of pulmonary fibrosis

Jack Leslie[1#], Ben JM Millar[1#], Alicia del Carpio Pons[1], Rachel A Burgoyne[1], Joseph D Frost[1], Ben S Barksby[1], Saimir Luli[1], Jon Scott[2], A John Simpson [2,3], Jack Gauldie[4], Lynne A Murray[5], Donna K Finch[5], Alan M Carruthers[5], John Ferguson[5], Matthew A Sleeman[5], David Rider[5], Rachel Howarth[1], Christopher Fox[1], Fiona Oakley[1], Andrew J Fisher[1,6], Derek A Mann[1], Lee A Borthwick[1]*

[1] Newcastle Fibrosis Research Group, Biosciences Institute, Newcastle University, Newcastle upon Tyne, UK.

[2] Biosciences Institute, Newcastle University, Newcastle upon Tyne, United Kingdom.

[3] Interstitial Lung Disease Clinic, Newcastle upon Tyne Hospitals NHS Foundation Trust, United Kingdom.

[4] St Joseph's Healthcare and Department of Pathology and Molecular Medicine, Firestone Institute for Respiratory Health, McMaster University Hamilton, Hamilton, Ontario, Canada.

[5] MedImmune Ltd., Cambridge, UK, CB21 6GH.

[6] Institute of Transplantation, Freeman Hospital, Newcastle upon Tyne, UK.

# authors contributed equally to this study

* Correspondence author

Dr Lee Borthwick

Newcastle Fibrosis Research Group

Biosciences Institute

4th Floor, William Leech Building

Newcastle University

Newcastle upon Tyne

NE2 4HH

Tel: 0191 208 3112

Email: lee.borthwick@ncl.ac.uk

Conflict of interest statement

DF and LM are employees of an AstraZeneca group company and may receive AstraZeneca shares as part of their usual remuneration. Work in the laboratory of LAB and DAM has been funded by AstraZeneca, GlaxoSmithKline and Abbvie. FO, DAM and LAB are co-founders, directors and shareholders of Fibrofind Ltd.

Figure S1- *fpr1-/-* mice are not protected from CCl_4 induced acute liver injury
A) C57Bl/6 and *fpr1-/-* mice were challenged with a single intraperitoneal dose of carbon tetrachloride (CCl_4) at 2µl/g body weight (CCl_4:olive oil at 1:1 [vol/vol]) or olive oil as a control. Serum and liver tissue were harvested at 24, 48 and 72 hours. Serum AST (**B**) and ALT (**C**) levels (U/L) were quantified. Number of **D)** NIMP and **E)** CD68 positive cells and **F)** PCNA positive hepatocytes per x20 magnification field. Data represent the mean value of n=20 randomly selected non-overlapping x20 magnification fields per mouse. **G)** Percentage area positive of α-smooth muscle actin (αSMA). Data represents the mean value of n=20 randomly selected, non-overlapping fields at x10 magnification. No significant difference was seen between olive oil-treated C57Bl/6 and *fpr1-/-* mice and therefore olive oil treated mice were pooled and presented as mean (red hashed line). n=5-8 mice per group. Data were analysed using a Mann-Whitney U test and presented as box and whiskers plots. All p-values >0.05.

Figure S2 - CCl_4-induced liver damage is comparable in C57Bl/6 and *fpr1-/-* mice
C57Bl/6 and *fpr1-/-* mice were challenged bi-weekly for 8 weeks with intraperitoneal injection of CCl_4 at 2µl/g body weight (CCl_4: olive oil at 1:3 [vol/vol]) or olive oil as a control. Mice were killed 24 hours after the last CCl_4 injection. Serum AST (**A**) and ALT (**B**) levels (U/L) were quantified. **C)** Number of PCNA positive cells per x20 magnification field. Data represent the mean value of n=20 randomly selected, non-overlapping fields per mouse. Relative gene expression of MMP-2 was assessed by qPCR **(D)**. Gene expression was normalised to GAPDH as a loading control. No significant difference was seen between olive oil-treated C57Bl/6 and *fpr1-/-* mice and therefore olive oil-treated mice are grouped and presented as mean (red hashed line). n=5-9 mice per group. Data were analysed using Mann-Whitney U test and presented as box and whiskers plots. All p-values >0.05.

Figure S3- *fpr1-/-* mice are not protected from MCD or BDL induced liver fibrosis
C57Bl/6 and *fpr1-/-* mice were fed a methionine/choline deficient diet (MCD) or control methionine/choline sufficient (MCS) diet and then harvested after 6 weeks. **A**) Representative αSMA stained liver sections in MCD fed mice. **B**) Serum aspartate aminotransferase (AST) and Alanine transaminase (ALT) levels expressed as units/litre (U/L). **C**) Relative gene expression of αSMA (i), Collagen 1A1 (ii), TGFβ1 (iii) and Timp1 (iv) in liver tissue was assessed by qPCR and normalised to GAPDH as a loading control. No significant difference was seen between MCS fed C57Bl/6 and *fpr1-/-* mice and therefore MCS-treated mice were pooled and presented as mean (red hashed line). C57Bl/6 and *fpr1-/-* mice also underwent bile duct ligation (BDL) and were harvested 10 days post-surgery. **D**) Representative αSMA stained liver section in BDL injured mice. **E**) Serum AST and ALT levels in BDL injured mice. **F**) Relative gene expression of αSMA (i), Collagen 1A1 (ii), TGFβ1 (iii) and Timp1 (iv) in liver tissue was assessed by qPCR and normalised to GAPDH as a loading control. No significant difference was seen between Sham C57Bl/6 and *fpr1-/-* mice and therefore sham mice were pooled and presented as mean (red hashed line). n=6-10 mice per group for panels A-F and n=5-9 mice per group for panels G-L. Data were analysed using a Mann-Whitney U test and presented as box and whiskers plots.

Gene target	Gene name	Forward primer sequence	Reverse primer sequence
Collagen 1a1	*Col1a1*	TTCACCTACAGCACGCTTGTG	GATGACTGTCTTGCCCCAAGTT
Fibronectin	*Fn1*	GAGCAAGAAGGACAACAGAG	GGTCTGGGGTTGGTAAATAG
Elastin	*Eln*	CCCTGTCCCTGTTCCTTCTG	CGCTCCCTATCCTCTTGTTG
MMP2	*Mmp2*	CCGATGCTGATACTGACACT	GTCACTGTCCGCCAAATAAA
KC	*Cxcl2*	CTGGGATTCACCTCAAGAACATC	CAGG-GTCAAGGCAAGCCTC
MCP-1	*Ccl2*	AGGTCCCTGTCATGCTTCTG	TCTGGACCCATTCCTTCTTG
GAPDH	*Gapdh*	GCACAGTCAAGGCCGAGAAT	GCCTTCTCCATGGTGGTGAA

Table S1. Primer sequences
